# Transplantation of mouse embryonic stem cell-derived oligodendrocytes in the murine model of globoid cell leukodystrophy

**DOI:** 10.1186/s13287-015-0024-2

**Published:** 2015-03-14

**Authors:** Xiao Ling Kuai, Run Zhou Ni, Guo Xiong Zhou, Zheng Biao Mao, Jian Feng Zhang, Nan Yi, Zhao Xiu Liu, Nan Shao, Wen Kai Ni, Zhi Wei Wang

**Affiliations:** Department of Gastroenterology, Nantong University Affiliated Hospital, 20 Xi Si Road, Nantong, Jiangsu 226001 China; Department of General Surgery, Nantong University Affiliated Hospital, 20 Xi Si Road, Nantong, Jiangsu 226001 China

## Abstract

**Introduction:**

Globoid cell leukodystrophy (GLD) is a severe disorder of the central and peripheral nervous system caused by the absence of galactocerebrosidase (GALC) activity. Cell-based therapies are highly promising strategies for GLD. In this study, G-Olig2 mouse embryonic stem cells (ESCs) were induced into oligodendrocyte progenitor cells (OPCs) and were implanted into the brains of twitcher mice, an animal model of GLD, to explore the therapeutic potential of the cells.

**Methods:**

The G-Olig2 ESCs were induced into OPCs by using cytokines and a multi-step differentiation procedure. Oligodendrocyte markers were detected by reverse transcription-polymerase chain reaction (RT-PCR) and immunocytochemistry. The toxicity of psychosine to OPCs was determined by a cell proliferation assay kit. The GALC level of OPCs was also examined. OPCs were labeled with Dir and transplanted into the brains of twitcher mice. The transplanted cells were detected by in-Vivo Multispectral Imaging System and real-time PCR. The physiological effects of twitcher mice were assessed.

**Results:**

Oligodendrocyte markers were expressed in OPCs, and 76% ± 5.76% of the OPCs were enhanced green fluorescent protein (eGFP)-positive, eGFP was driven by the Olig2 promoter. The effect of psychosine on cell viability indicated that OPCs were more resistant to psychosine toxicity. The GALC level of OPCs was 10.0 ± 1.23 nmol/hour per mg protein, which was significantly higher than other cells. Dir-labeled OPCs were injected into the forebrain of post-natal day 10 twitcher mice. The transplanted OPCs were myelin basic protein (MBP)-positive and remained along the injection tract as observed by fluorescent microscopy. The level of the Dir fluorescent signal and eGFP mRNA significantly decreased at days 10 and 20 after injection, as indicated by in-Vivo Multispectral Imaging System and real-time PCR. Because of poor cell survival and limited migration ability, there was no significant improvement in brain GALC activity, MBP level, life span, body weight, and behavioral deficits of twitcher mice.

**Conclusions:**

ESC-derived OPC transplantation was not sufficient to reverse the clinical course of GLD in twitcher mice.

## Introduction

Globoid cell leukodystrophy (GLD), or Krabbe disease, is an autosomal recessive disease caused by the deficiency of galactocerebrosidase (GALC) activity, which is involved in the metabolism of galactosylceramide and psychosine [1,2]. Psychosine is a toxic metabolite that accumulates in GLD and results in degeneration and apoptosis of oligodendrocytes, causing demyelination of the central nervous system (CNS) and peripheral nervous system [3].

Cell-based therapies are highly promising strategies for neurodegenerative diseases. In addition to oligodendrocyte progenitors (OPCs), Schwann cells and olfactory ensheathing cells (OECs) have been explored as donor sources for cell transplantation therapy [4-6]. The clinical application of OPCs and OECs is hampered by the limited access to primary cells derived from the CNS. Neural stem cells (NSCs) and oligodendroglial cell lines have been considered as alternative therapeutic avenues [7-9]. The isolation of these cells also requires obtaining CNS tissue. The oligodendroglial differentiation of bone marrow-derived adult stem cells has been described *in vitro* and *in vivo* by many investigators; however, an unambiguous demonstration of adult stem cell differentiation into functional oligodendroglial cells has still not been established [10-12].

Embryonic stem cells (ESCs) have the potential to generate cells of all three embryonic germ layers [13,14], and many studies have shown the *in vitro* differentiation of ESCs into various cell types [15-18], including neural lineage cells [19-22]. Because of their self-renewal capacity and pluripotency, ESCs provide novel prospects for cellular replacement strategies for neural degenerative diseases, including GLD.

The twitcher mouse is an animal model for human GLD (Krabbe disease). Twitcher mice have a spontaneous recessive mutation of the lysosomal enzyme galactocerebroside beta-galactosidase (GALC), which blocks the catabolism of galactosylceramide (or galactocerebroside) and results in an accumulation of the cytotoxic substrate of the enzyme GALC, and psychosine, which causes the death of myelin-forming cells (oligodendrocytes and Schwann cells) and demyelination [23]. The twitcher mouse is considered to be a valuable model for clinical trials for the treatment of Krabbe disease.

In twitcher mice, bone marrow transplantation has been the only therapeutic approach that significantly delays disease onset and progression and can potentially deliver the functional enzyme GALC to the CNS by macrophage/microglia replacement with donor-derived cells [24]. Previous studies have indicated that NSC/progenitor cell types engrafted in the twitcher mouse brain have therapeutic benefit, in which the engrafted cells secrete the GALC enzyme. However, important issues, such as the long-term survival of NSCs in the toxic environment and the efficacy of NSC transplants, remain controversial [25,26].

In this study, mice ESCs were induced to differentiate along oligodendrocytic lineages. The therapeutic potential of ESC-derived oligodendrocytes in twitcher mice was investigated. The cells were injected into the forebrain of twitcher mice on post-natal day (PND) 10. Life span, weight, twitching frequency/severity, and motor function were recorded. The brain tissues of the mice were collected to analyze myelin, survival, differentiation, and migration of engrafted cells. We also monitored transplanted cells with an *in vivo* imaging system.

## Methods

### Cell culture

The G-Olig2 ESC line (SCRC-1037; ATCC, Manassas, VA, USA), was cultured as described [27]. The G-Olig2 ESC line is a knock-in ESC line created by the insertion of the enhanced green fluorescent protein (eGFP) cDNA into the Olig2 gene. Therefore, eGFP expression was detected only in cells with Olig2 gene expression. Undifferentiated ESCs were maintained on feeder-free, gelatin-coated plates in the ESC growth medium containing Dulbecco’s modified Eagle’s medium (DMEM), 15% knockout serum replacement, 1× non-essential amino acids, sodium pyruvate (1 mM), sodium bicarbonate (0.075%), L-glutamine (1 mM), 2-mercaptoethanol (0.1 mM), and human recombinant leukemia inhibitory factor (LIF) (1,000 units/mL) (all reagents from Invitrogen, Rockville, MD, USA).

TwS1, a spontaneously immortalized twitcher mouse Schwann cell line, was obtained from Watabe Kazuhiko at Jikei University School of Medicine, Tokyo, Japan. The TwS1 cells were cultured in DMEM with 10% fetal bovine serum (Hyclone, Logan, UT, USA) in accordance with the instructions of Watabe and colleagues [28].

### Inducing G-Olig2 embryonic stem cells into oligodendrocyte

**Figure 1:**
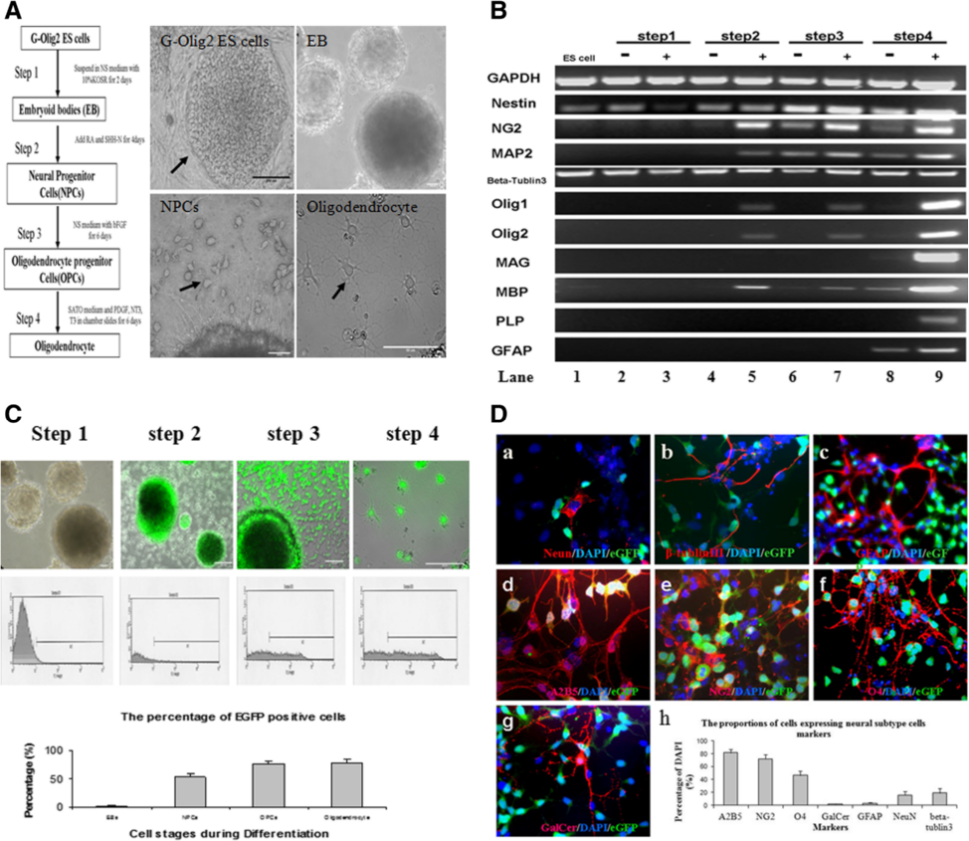
**Differentiation of G-Olig2 embryonic stem cells (ESCs) into oligodendrocytes. (A)** Differentiation procedures and cell morphology of each step: (upper left frame) G-Olig2 ESC colony, (upper right) embryoid bodies (EBs), (lower left) neural lineage cells (arrow), and (lower right) oligodendrocyte-like cells (arrow). **(B)** RT-PCR results. Nestin, MAP2, and β-tublin III are markers for neurons. GFAP for astrocytes. NG2, Olig1, Olig2, MAG, myelin basic protein (MBP), and PLP are oligodendrocyte markers. Minus sign (−) indicates spontaneously differentiated cells; plus sign (+) indicates induced cells. Step 1 (EBs), step 2 (NPCs), step 3 (OPCs), and step 4 (oligodendrocytes) are shown. In lane 1, nestin and β-tublin III are expressed in undifferentiated G-Olig2. In lanes 2 and 3 (EBs), nestin and β-tublin III are expressed. In lanes 4 and 5 (NPCs), NG2, MAP2, Olig1, Olig2, and MBP transcription factors are induced. In lanes 6 and 7 (OPCs), nestin, β-tublin III, NG2, MAP2, Olig1, Olig2, and MBP are expressed. In lanes 8 and 9 (oligodendrocyte), oligodendrocyte-specific genes MAG, MBP, and PLP are expressed at the terminal stage. Nestin, β-tublin III, NG2, MAP2, Olig1, Olig2, and GFAP genes are also expressed. **(C)** eGFP expression-positive cells in each step was detected by fluorescence microscopy, and the percentage was analyzed through fluorescence-activated cell sorting. Step 1 (EBs): only sporadic eGFP-positive cells in the EBs. Step 2 (NPCs): 52.59% ± 6.58% of NPCs was eGFP-positive. Step3 (OPCs) and Step4 (oligodendrocytes): 76% ± 5.76% of OPCs and 78.4% ± 5.95% of oligodendrocytes were eGFP-positive. **(D)** Immunostaining of terminal differentiated cells: (a) (NeuN)-positive cells, (b) β-tublin III-positive cells, (c) GFAP-positive cells, (d) A2B5-positive cells, (e) NG2-positive cells, (f) O4-positive cells, (g) GALC-positive cells, and (h) the percentage of positive cells. The secondary antibodies are labeled with rhodamine (red). nuclei were stained with DAPI (blue).

G-Olig2 ESCs were induced into oligodendrocytes as described [27]. Briefly, the sequential culture procedure included embryoid body (EB) formation (step 1), induction of neural progenitor cells (NPCs) from EBs (step 2), expansion and differentiation of NPCs into oligodendrocyte progenitor cells (OPCs) (step 3), and differentiation of OPCs along oligodendrocyte lineage (step 4) (Figure 1A).

ESCs were trypsinized, and 1 × 10^6^ cells were plated onto 10-cm Petri dishes (Fisher, Hampton, NH, USA) in suspension to form EBs with neural differentiation medium (NS medium) containing DMEM, neurobasal medium, 1 × N2 supplement, L-glutamine (1 mM), 2-mercaptoethanol (0.1 mM), and 10% knockout serum replacement (all reagents from Invitrogen). Two days later, retinoic acid (RA, 0.2 μM; Sigma-Aldrich, St. Louis, MO, USA) and recombinant SHH-N (1 μg/mL; R&D Systems, Minneapolis, MN, USA) were added simultaneously to the (NS) medium to promote NPCs differentiation. After 4 days, NPCs were induced into OPCs by adding fibroblast growth factor 2 (FGF2) (20 ng/mL; Sigma-Aldrich) and heparin (2 μg/mL; Stemcell Technologies, Vancouver, BC, Canada) in the NS medium, and the cells were cultured for an additional 6 days. The cell aggregates (OPCs) were dissociated with a NeuroCult Chemical Dissociation Kit (Stemcell Technologies), and 5 × 10^4^ cells were plated onto poly-L-lysine- and laminin-coated chamber slides (Becton Dickinson, Franklin Lakes, NJ, USA) in a modified Bottenstein-Sato medium with platelet-derived growth factor AA (PDGF-AA) (10 ng/mL; R&D Systems), T3 (40 μg/mL; Sigma-Aldrich), and NT3 (10 ng/mL, Sigma-Aldrich) to promote oligodendrocyte differentiation. The modified Bottenstein-Sato medium contained B27 (Invitrogen), N-acetyl-cysteine (60 μg/mL), putresine (16 μg/mL), biotin (10 μg/mL), and cAMP (1.6 μg/mL) (all reagents were from Sigma-Aldrich).

### Psychosine treatment assay

Psychosine (Sigma-Aldrich) was dissolved in ethanol at a concentration of 20 mM and was further diluted in DMEM to indicate concentrations. To investigate the effect of psychosine on cell viability, TwS1 (an immortalized twitcher mouse Schwann cell line), OPCs, and terminally differentiated oligodendrocytes (1.2 × 10^5^/cm^2^) were cultured in DMEM for 24 hours and then treated with 10 to 80 μM psychosine. The cell viability was assessed 48 hours after psychosine treatment.

### Viability assay

Cell viability was determined with the Cell Proliferation Assay Kit (Chemicon, Temecula, CA, USA) in accordance with the recommendations of the manufacturer. This procedure was based on the cleavage of the tetrazolium salt WST-1 to formazan by cellular mitochondrial dehydrogenases. More viable cell results in an increase in the overall activity of the mitochondrial dehydrogenases. The augmentation in the enzyme activity leads to an increase in the amount of formazan dye that is formed. The formazan dye, produced by viable cells, can be quantified with a multi-well spectrophotometer by measuring the absorbance of the dye solution at 450 nm.

### Flow cytometry

G-Olig2 ESCs were prepared for fluorescence-activated cell sorting (FACS) analysis of eGFP expression at each differentiation step. Cells or cell aggregates were dissociated by trypsinization or chemical dissociation. DMEM containing 10% bovine serum was added to quench the trypsin. The cells were then washed and suspended in phosphate-buffered saline (PBS). eGFP-positive cells were analyzed on a flow cytometer within 1 hour after sample preparation. Cells were also viewed under a Nikon Eclipse E600 fluorescence microscope (Nikon, Tokyo, Japan).

### Reverse transcription-polymerase chain reaction

The induction of the expression of mRNAs for oligodendrocyte lineage-specific genes was assessed at each step of differentiation. Table 1 lists the sequence of all of the polymerase chain reaction (PCR) primers and the sizes of PCR products. ESCs cultured in ESC growth medium without LIF were used as negative control samples. Total cellular RNA was isolated by using a RNeasy Mini Kit (Qiagen, Valencia, CA, USA) and treated with a DNA-free kit (Ambion, Austin, TX, USA) to remove potential contamination of genomic DNA. A total of 500 ng of RNA was used as a template for reverse transcription with Reverse Transcription System (Promega, Madison, WI, USA). After the RT step, the concentration of the cDNA was adjusted to the same level, and 100 ng of cDNA was used for a standard PCR for the described primer sets. A housekeeping gene, glyceraldehyde-3-phosphate dehydrogenase (GAPDH), was used as a control for the PCR efficiency of each sample. The PCR step was performed by using a PCR Master Mix kit (Promega), and the PCR products were detected and analyzed by 2% agarose gel electrophoresis.

**Table 1:** **List of primers used for reverse transcription-polymerase chain reaction**

**Gene name**	**Primer sequence**	**Product size, base pairs**
*GAPDH*	5′-ACC-ACA-GTC-CAT-GCC-ATC-AC-3′	450
	5′-TCC-ACC-ACC-CTG-TTG-CTG-TA-3′	
*NG2*	5′-AGA-AGA-CCC-GCA-GGC-TCA-AG-3′	338
	5′-CGT-GGA-GTT-GGA-GGA-TGA-CG-3′	
*Olig1*	5′-AAG-GAG-GAC-ATT-TCC-AGA-CTT-C-3′	154
	5′-GCT-CTA-AAC-AGG-TGG-GAT-TCA-TC-3′	
*Olig2*	5′-TCA-TCT-TCC-TCC-AGC-ACC-TC-3′	305
	5′-CCG-TAG-ATC-TCG-CTC-ACC-AG-3′	
*Nestin*	5′-AAC-TGG-CAC-ACC-TCA-AGA-TGT-3′	235
	5′-TCA-AGG-GTA-TTA-GGC-AAG-GGG-3′	
*GFAP*	5′-CAC-GAA-CGA-GTC-CCT-AGA-GC-3′	234
	5′-ATG-GTG-ATG-CGG-TTT-TCT-TC-3′	
*MAP2*	5′-CTG-GAC-ATC-AGC-CTC-ACT-CA-3′	164
	5′-AAT-AGG-TGC-CCT-GTG-ACC-TG-3′	
*MAG*	5′-CGG-AGA-GGG-AGT-TTG-TGT-ACT-CCG-3′	530
	5′-CTC-CTC-TGT-CAG-GGT-GTA-GCT-GTC-3′	
*MBP*	5′-GTC-ACC-ATC-TCT-CCT-CAG-TGG-CTC-3′	350
	5′-GTT-CTC-AGC-TCC-TCA-TCC-CTG-GAG-3	
*PLP*	5′-CGA-CTA-CAA-GAC-CAC-CAT-CTG-CGG-3′	302
	5′-CAG-CGC-AGA-GAC-TGC-CTA-TAC-TGG-3′	
*Beta-tublin III*	5′-GAC-TCA-GTC-CTA-GAT-GTC-GTG-CGG-3′	388
	5′-GGA-ATC-GAA-GGG-AGG-TGG-TGA-CTC-3′	

### Transplantation

Twitcher mice (GALC^twi/+^) were obtained from Jackson Laboratories (Bar Harbor, ME, USA). All experiments were performed according to the Institutional Guidelines for Animal Care and Use and were approved by the Animal Experimentation Ethics Committee of Nantong University affiliated Hospital. Before cell transplantation, OPCs were incubated with 5 μg/mL cellular labeling dye DiR (D12731; Invitrogen) at 37°C for 1 hour. Cells were then washed twice with PBS to remove excess dye. Subsequently, OPCs were dissociated with the NeuroCult Chemical Dissociation Kit (Stemcell Technologies) into single cells. The cells were counted by Trypan Blue exclusion (viability of more than 90%) and resuspended (10,000 cells/μL) in PBS with 0.1% DNase (Sigma-Aldrich, St. Louis, MO, USA). Cells were kept in ice before injection.

Animals were briefly anesthetized on wet ice and injected with OPCs into the forebrain on PND 10 by using these stereotaxic coordinates: 1.5 mm lateral and 0.2 mm posterior to bregma and depth of 2 mm. The twitcher mice (GALCt^wi/twi^) received bilateral OPC injections, 1 μL per hemisphere using a Hamilton syringe. The twitcher mice (GALC^twi/twi^) received the same volume of saline as the controls.

### Tissue processing

The twitcher mice lived until the terminal stage (body weight of less than 80% than age-matched wild-type mice or inability to eat and drink) and then were killed. Mice were perfused with saline (PBS) to remove contaminating blood, followed by 4% paraformaldehyde (PFA). Brains were then post-fixed with 4% PFA overnight at 4°C and then cryoprotected with 30% sucrose. The brains were cut into 2-mm wide blocks and then flash-frozen in optimal cutting temperature (OCT) embedding medium by using liquid nitrogen and stored at −80°C. Cryosections of 16-μm thickness were cut from each block and used for immunohistochemistry.

### Real-time polymerase chain reaction

To track eGFP^+^ cells in the brain of twitcher mice, DNA was extracted from the brain tissue of the mice at different time points after cell injection with a DNeasy Blood and Tissue Kit (Qiagen, Valencia, CA, USA). DNA was examined for the presence of the eGFP gene by using real-time PCR by the standard curve method for absolute quantification with Sybr Green (Applied Biosystems, Foster City, CA, USA) by using the following primers: forward 5′-CAG AAG AAC GGC ATC AAG GTG-3′ and reverse 5′-TGG GTG CTC AGG TAG TGG TTG-3′. The primers for myelin basic protein (MBP) were forward 5′-GGC CTC AGA GGA CAG TGA TG-3′ and reverse 5′-TCT GCT GTG TGC TTG GAG TC-3′. The primers for GAPDH were forward 5′-CGT CCC GTA GAC AAA ATG GT-3′ and reverse 5′-TTG ATG GCA ACA ATC TCC AC-3′. An AB 7900HT Real-Time sequence analyzer (Applied Biosystems) was applied. Relative quantification of eGFP and MBP expression was calculated using the comparative cycle threshold (CT) method. The relative values of eGFP and MBP expression were normalized to the endogenous housekeeping gene, GAPDH, and calculated relative expression values. Data were presented as the mean value ± standard deviation (SD).

### Galactocerebrosidase level test

TwS1, OPCs, and terminally differentiated oligodendrocytes were harvested from cell culture dishes. Fresh brain tissue was collected from mice killed by CO_2_. The tissue and cells were homogenized in four volumes of 20 mM acetate buffer (pH 4.5). The substrate solution for GALC, containing oleic acid, sodium taurocholate, triton-X-100, and [3H]GalCer, was prepared as previously published [29]. Radioactive [3H]GalCer, the substrate for GALC, was cleaved into [3H]galactose and ceramide by GALC. Brain and cell homogenate (50 μL) was added to 50 μL of the substrate solution and incubated at 37°C for 8 hours. The reaction was stopped by the addition of 5 μL of chloroform-methanol (2:1), 0.1 mL of 1 mg/mL galactose, and 0.8 mL of water. The mixture was centrifuged at 3,000 revolutions per minute for 10 minutes; the radioactivity in 0.9 mL of the aqueous phase was measured with a scintillation counter (TriCarb Model 1600CA; Packard, DownersGrove, IL, USA). GALC activity is expressed as nanomoles of [3H]GalCer hydrolyzed per hour per milligram of protein.

### Immunocytochemistry

Cells at the last step of differentiation were cultured in chamber slides and then were fixed in 4% PFA in PBS for 15 minutes, permeabilized with 0.1% Triton X-100 for 10 minutes, and then blocked for 1 hour at room temperature in PBS containing 5% goat serum (Invitrogen). Samples were then incubated in blocking buffer containing primary antibody for 2 hours at room temperature and washed three times with PBS for 15 minutes. For staining of NG2 and O4, unfixed viable cells were first stained with primary antibodies for 30 minutes and then fixed with 4% PFA in PBS for 15 minutes. Afterwards, the cells were permeabilized with 0.1% Triton X-100 and blocked for 1 hour at room temperature with the blocking buffer. The staining procedure for all of the secondary antibodies was identical. Cells were incubated with secondary antibodies conjugated with Texas Red (1:1,000; Molecular Probes, Eugene, OR, USA) for 1 hour at room temperature. The samples were washed as above and mounted with 6-diamidino-2-phenylindole (DAPI) (Dako, Carpinteria, CA, USA) containing mounting solution. The following primary mouse antibodies were used: anti-NeuN (IgG, 1:100), anti-A2B5 (IgM, 1:100), anti-β tubulin III (IgG, 1:50), anti-GFAP (IgG, 1:100), anti-O4 (IgG, 1:100), anti-galactocerebroside (IgG, 1:100) and anti-MBP (IgG, 1:50). The antibody anti-NG2 is a polyclonal antibody from rabbit (1:200). All primary antibodies were obtained from Millipore (Billerica, MA, USA). Fluorescent samples were examined with a Nikon Eclipse E600 fluorescence microscope (Nikon, Tokyo, Japan).

The cryosections of brains were washed with PBS containing fish skin gelatin (FSG) (G-7765; Sigma-Aldrich, St. Louis, MO, USA) and Triton x-100 (Sigma-Aldrich) for 30 minutes at room temperature. Sections were blocked with 10% normal goat serum (Invitrogen) in PBS-FSG for 1 hour at room temperature and then with anti-MBP (1:50, MAB386; Millipore) for 1 hour at room temperature. Sections were then washed twice with PBS-FSG-Tx100 and then once with PBS-FSG for 10 minutes each. Sections were then incubated with secondary antibody goat anti-mouse-Alexa 568 (1:1,000; Invitrogen) for 1 hour at room temperature, washed, and mounted with coverslips for fluorescence microscope evaluation. Sections were always prescreened for the presence of eGFP before immunohistochemistry was performed.

### Western blotting

The brain homogenate in acetate buffer was centrifuged at 10,000 *g* for 15 minutes to obtain a cleared lysate. A total of 20 μg of protein from each sample was loaded onto a 4% to 20% PAGE and then transferred to a polyvinylidene difluoride (PVDF) membrane. Membranes were incubated with anti-MBP (1:2,500, MAB386; Millipore) overnight at 4°C. Membranes were probed with anti-GAPDH (1:500, ab9485; Abcam) overnight at 4°C for normalization. The grayscale of the bands was measured and the relative quantitative value of MBP to GAPDH was analyzed with Gel-Pro-analyzer software. Data were presented as the mean value ± SD.

### *In vivo* distribution of the transplanted cells

To look for GFP-positive cells in the brain, the tissue sections were mounted on a slide, and nuclei were counterstained with a DAPI-containing mounting medium (Dako) and examined with a fluorescence microscope. The location and the fluorescent strength of the transplanted cells labeled with dye DiR were detected by the Kodak In-Vivo Multispectral Imaging System FX (Kodak, Rochester, NY, USA) at different time points on days 1, 10, and 20. To perform this evaluation, mice were anaesthetized by intraperitoneal injection of a ketamine (80 mg/kg) + xylazine (16 mg/kg) mixture and then imaged. At the end of each acquisition, a photographic image was obtained. The data were analyzed with Photovision software (Kodak, Rochester, NY, USA), which superimposes the signal on the photographic image. The most intense bioluminescence signal detected is shown in red, whereas the weakest signal is shown in blue.

### Assessment of physiological effects

The twitcher mice lived until terminal stage (body weight of less than 80% that of age-matched wild-type mice or inability to eat and drink) and were killed. The life span of the twitcher mice was measured by the date of killing. Body weight was measured every 3 days beginning on PND 17 until the date of killing. Twitching frequency and severity were also scored every 3 days beginning on PND 17 by using the following scoring system: frequency—rare (1), intermittent (2), and constant (3); severity—fine (1), mild (2), moderate (3), and severe (4) [30]. The hind stride length of both the left and right back paws was measured and averaged together.

### Statistical analysis

The data were expressed as the mean ± SD. The statistical significance was assessed with Student’s *t* test between two groups. The log-rank test was performed for Kaplan-Meier survival curve analysis. *P* values below 0.05 were considered statistically significant.

## Results

### The characterization of G-Olig2 embryonic stem cell-derived oligodendrocyte

G-Olig2 ESCs were induced into oligodendrocyte with a multi-step differentiation protocol (Figure 1A). G-Olig2 ESCs were cultured on feeder-free, gelatin-coated plates in the ESC growth medium in an undifferentiated state (Figure 1A). The expression of nestin and β-tublin III mRNAs (Figure 1B, lane 1) in undifferentiated G-Olig2 ESCs was positive. EGFP expression in undifferentiated G-Olig2 ESCs was undetectable (data not shown).

The first step of differentiation was EB formation (Figure 1A). At this step, still only nestin and β-tublin III transcription factors were expressed (Figure 1B, lane 3). The eGFP expression was 2.3% ± 0.51% (Figure 1C, step 1).

After adding RA and SHH-N to the NS medium for 4 days (step 2), the percentage of eGFP-positive cells increased to 52.59% ± 6.58% (Figure 1C, step 2), and NG2, MAP2, Olig1, Olig2, and MBP transcription factors started to be expressed in these cells (Figure 1B, lane 5).

Cells with branches projecting from small cell bodies were found at the edge of EBs after being cultured in NS medium with basic fibroblast growth factor for an additional 6 days (Figure 1A). The percentage of eGFP-positive cells was 76% ± 5.76% (Figure 1C, step 3). Reverse transcription-PCR (RT-PCR) results indicated that nestin, β-tublin III, NG2, MAP2, Olig1, Olig2, and MBP were expressed in these cells (Figure 1B, lane 7).

The cell aggregates were dissociated with a chemical dissociation kit and plated onto poly-L-lysine- and lamin-coated chamber slides in a modified Bottenstein-Sato medium with PDGF, NT3, and T3. After 6 days, cells with complex patterns of branches were found in the cell culture (Figure 1A). The percentage of EGFP-positive cells was 78.4% ± 5.95% (Figure 1C). Oligodendrocyte-specific genes, such as MAG, MBP, and PLP, were highly expressed at the terminal stage of differentiation (Figure 1B, lane 9). Nestin, β-tublin III, NG2, MAP2, Olig1, Olig2, and GFAP genes were also expressed in these cells (Figure 1B, lane 9). The terminally differentiated cells were stained with a panel of neural subtype-specific antibodies. The differentiated cells stained positively for oligodendrocyte precursor cell markers A2B5 (81.37% ± 5.06% of DAPI) and NG2 (72% ± 6.32% of DAPI) (Figure 1D-d, e, and h). Some cells expressed oligodendrocyte marker O4 (46.63% ± 6.05% of DAPI) and GalCer (1.5% ± 0.5% of DAPI) (Figure 1D-f, g, and h), and MBP was negative in the differentiated cells (data not shown). A few of the differentiated cells were reactive for neuron marker NeuN (15.93% ± 5.95% of DAPI), β-tublin III (18.97% ± 7.01% of DAPI), and GFAP (2.97% ± 1.45% of DAPI) for neural progenitors and astrocyte (Figure 1D-a, b, c, and h).

### Effects of psychosine on cell viability

**Figure 2:**
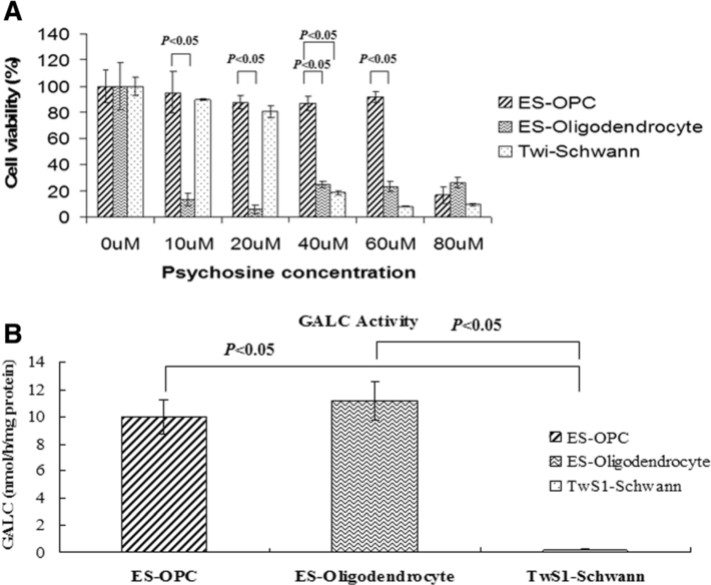
**Effects of psychosine on cell viability and galactocerebrosidase (GALC) activity. (A)** TwS1 cells, oligodendrocyte progenitor cells (OPCs), and terminally differentiated oligodendrocytes were treated with psychosine at concentrations varying from 10 to 80 μM. Compared with TwS1 cells and terminally differentiated oligodendrocytes, OPCs had a higher threshold for psychosine toxicity. The cell viability of OPCs decreased dramatically at 80 μM. **(B)** GALC activity was measured in TwS1, OPCs, and terminally differentiated oligodendrocytes. The GALC level of OPCs and terminally differentiated oligodendrocytes was significant higher than that of TwS1, and the differences were statistically significant (both *P* <0.05). There was no difference between OPCs and terminally differentiated oligodendrocytes in GALC level (*P* >0.05). ES, embryonic stem.

TwS1 cells, OPCs, and terminally differentiated oligodendrocytes were treated with psychosine at concentrations varying from 10 to 80 μM. The data showed that increasing the psychosine concentration resulted in a decrease in cell viability and that the cell viability varied in different types of cells (Figure 2). At 10 μM, the viability of terminally differentiated oligodendrocytes was 33.63% ± 6.8%, of OPCs was 95.15% ± 15.9%, and of TwS1 was 89.72% ± 0.72% (Figure 2A). At 40 μM, the cell viability of OPCs (87.625% ± 5.17%) was significantly higher than that of TwS1 cells (23.16% ± 4.6%) (*P* <0.05) (Figure 2A). At 60 μM, the cell viability of OPCs was still high (91.62% ± 4.16%) (Figure 2A). The cell viability of OPCs decreased dramatically at 80 μM (16.65% ± 6.47%) (Figure 2A). Because OPCs had a higher threshold for psychosine toxicity, OPCs were selected as the cell source for injection in the brains of twitcher mice.

### Galactocerebrosidase activity

GALC activity was measured in TwS1, OPCs, and terminally differentiated oligodendrocytes. The GALC levels of OPCs, TwS1, and terminally differentiated oligodendrocytes were 10.0 ± 1.23, 0.18 ± 0.05, and 11.2 ± 1.4 nmol/hour per mg protein, respectively (Figure 2B). The GALC level of OPCs and terminally differentiated oligodendrocytes was significant higher than that of TwS1, and the differences were statistically significant (both *P* <0.05) (Figure 2B). There was no difference between OPCs and terminally differentiated oligodendrocytes in GALC level (*P* >0.05) (Figure 2B).

GALC activity was almost undetectable in twitcher mice (GALC^twi/twi^) brains. There was no increase in GALC activity in the brains of twitcher mice (GALC^twi/twi^) that received OPC injection (0 nmol/hour per mg protein, *P* >0.05), although these cells exhibited an increase in GALC activity compared with twitcher cells *in vitro*.

### Presence of myelin

**Figure 3:**
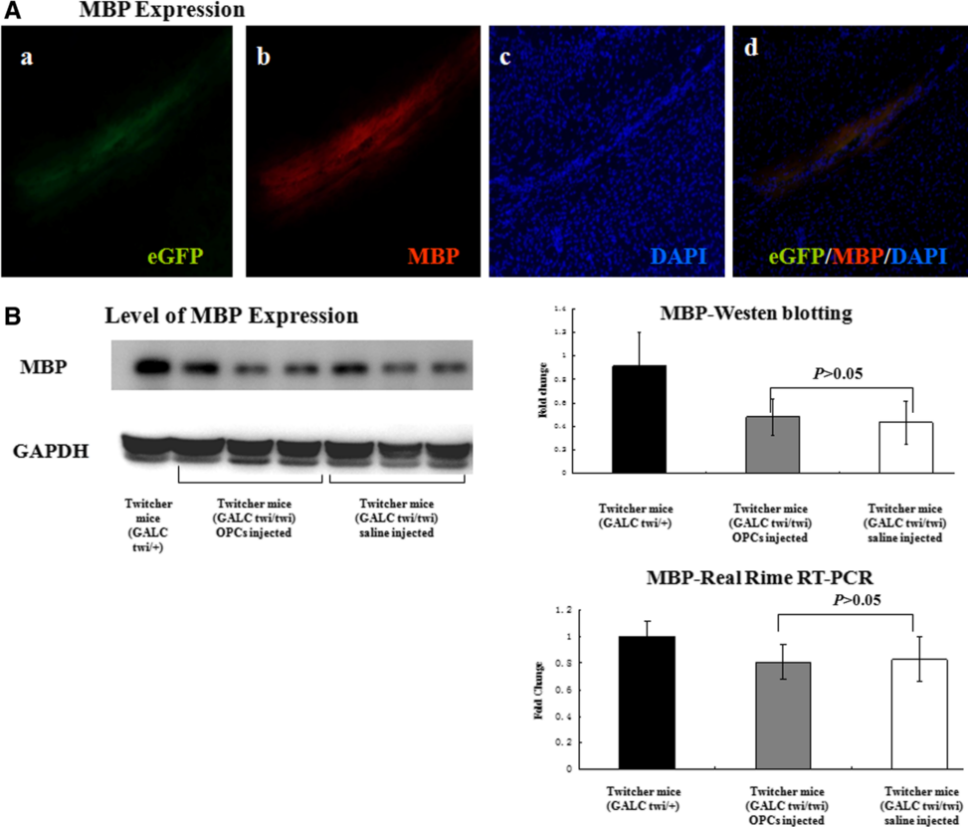
**Myelin basic protein (MBP) expression. (A)** The enhanced green fluorescent protein (eGFP)-positive cells were MBP-positive and located along the injection tract. (a) eGFP-positive cells (green), (b) MBP labeled with rhodamine (red), (c) nuclei were stained with 6-diamidino-2-phenylindole (DAPI) (blue), (d) merge. **(B)** The levels of MBP in the brain of twitcher mice were detected by Western blotting and real-time reverse transcription-polymerase chain reaction (RT-PCR). The results indicated that there was no significant difference in the level of MBP expression between the injection of oligodendrocyte progenitor cells (OPCs) and saline in twitcher mice (Western blotting: 0.478 ± 0.157 versus 0.432 ± 0.186; real-time RT-PCR: 0.81 ± 0.13 versus 0.83 ± 0.17; both *P* >0.05). GALC, galactocerebrosidase; GAPDH, glyceraldehyde-3-phosphate dehydrogenase.

The eGFP-positive cells were located along the injection tract under fluorescent microscopy in cryosections of the brains 20 days after injection (Figure 3A-a). The implanted cells were MBP-positive (Figure 3A-b). The levels of myelin in the brain of twitcher mice were detected by Western blotting and real-time RT-PCR at day 20 after cell injection. The results of Western blotting indicated that there was no significant difference in the level of myelin expression between the injection of OPCs (0.478 ± 0.157) and saline in twitcher mice (0.432 ± 0.186) (*P* >0.05) (Figure 3B). The results of real-time RT-PCR also showed that the injection of OPCs did not increase the levels of myelin in the twitcher brain (0.81 ± 0.13 versus 0.83 ± 0.17) (Figure 3B).

### *In vivo* distribution of the transplanted cells

**Figure 4:**
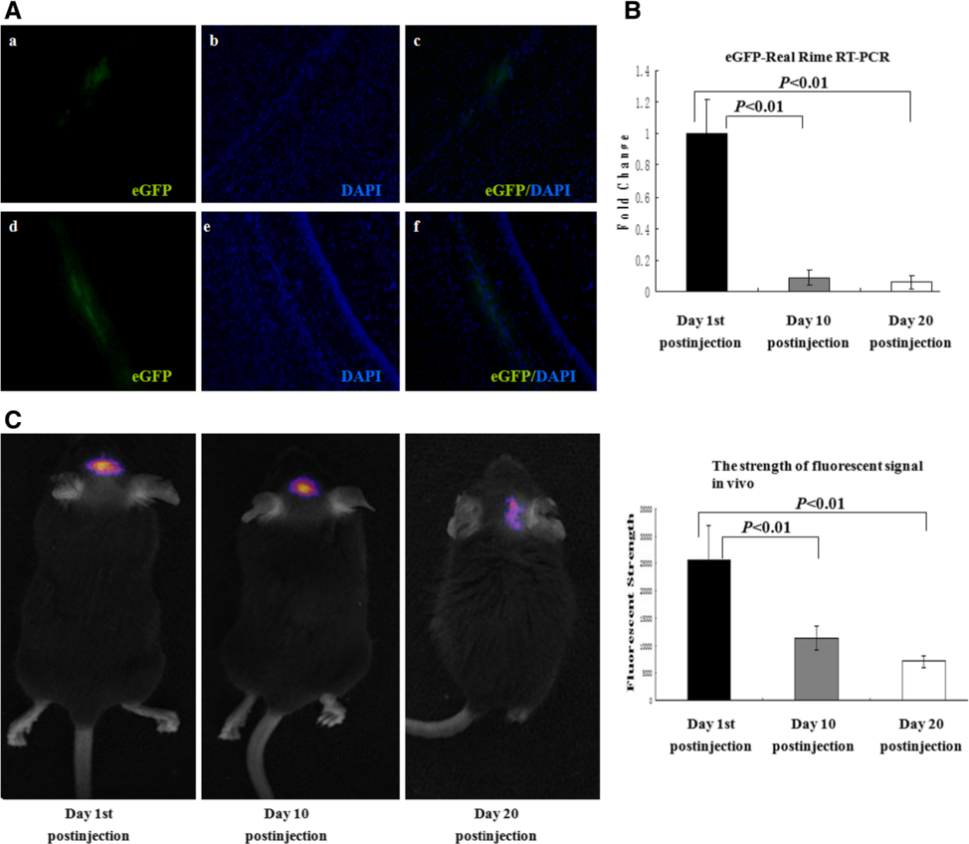
**Distribution of the transplanted cells in twitcher mice brain. (A)** The enhanced green fluorescent protein (eGFP)-positive cells were located along the injection tract: (a, d) eGFP-positive cells (green), (b, e) nuclei were stained with 6-diamidino-2-phenylindole (DAPI) (blue), (c, f) merge. **(B)** Real-time reverse transcription-polymerase chain reaction (RT-PCR) results of eGFP at different time points after injection. Compared with day 1 after injection, the expression level of eGFP significantly decreased at days 10 and 20 (0.09 ± 0.05 and 0.06 ± 0.04 versus 0.96 ± 0.22, both *P* <0.01). **(C)** Dir-labeled cells in twitcher mice brains were detected by in-Vivo Multispectral Imaging System. The strengths of fluorescent signal at days 1, 10, and 20 after injection were 25,522 ± 6,287, 11,372 ± 2,162, and 7,077 ± 1,022, and the differences were statistically significant (all *P* <0.01).

Dir-labeled and eGFP-positive OPCs were injected into the brains of twitcher mice. The eGFP-positive cells were detected in cryosections of the brains 20 days after injection (Figure 4A). The cells were found to be integrated and remained along the injection tract under fluorescent microscopy (Figure 4A).

The eGFP-positive cells were also tracked with real-time PCR at different time points after injection. The eGFP could be detected 20 days after injection; however, the expression level significantly decreased at days 10 and 20 after injection compared with day 1 after injection (0.09 ± 0.05 and 0.06 ± 0.04 versus 0.96 ± 0.22) (both *P* <0.01, Figure 4B).

A clear fluorescent signal was observed in mice analyzed at days 1 and 10 after implantation but was rarely seen at day 20 after implantation by in-Vivo Multispectral Imaging System. The strength of fluorescent signal significantly decreased at day 10 (11,372 ± 2,162) and day 20 (7,077 ± 1,022) after injection compared with day 1 (25,522 ± 6,287) after injection (*P* <0.01, Figure 4C).

### Life span, body weight, and physiological effects

**Figure 5:**
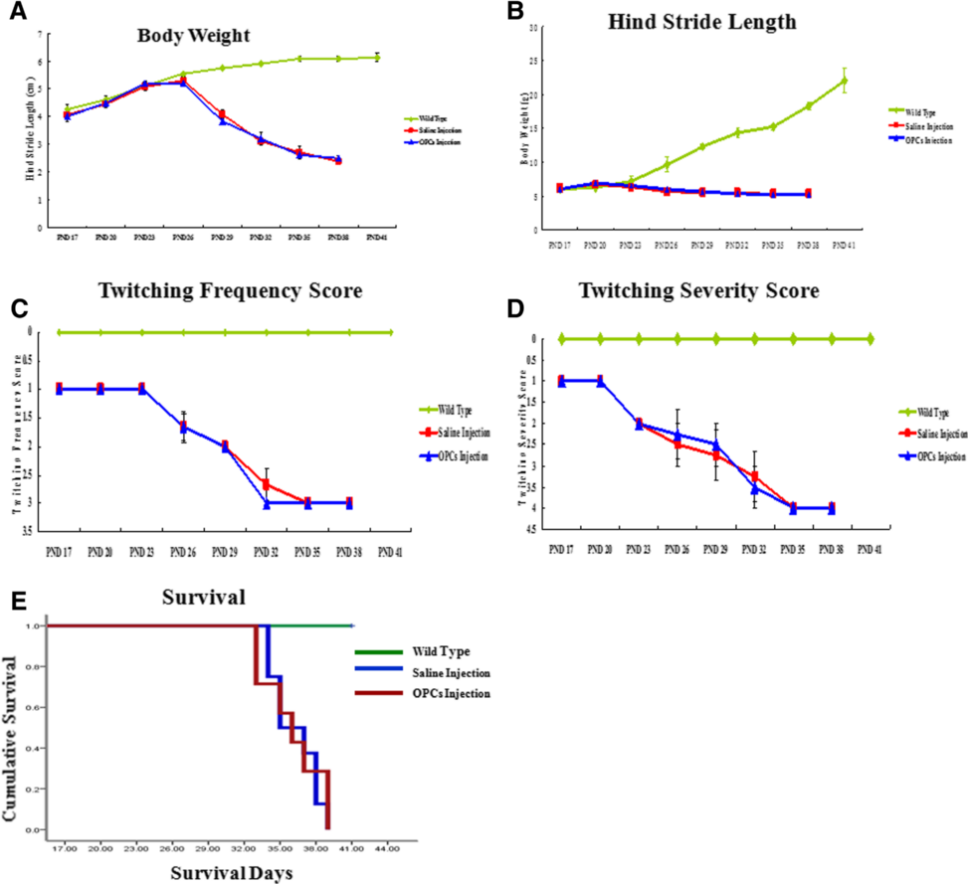
**Body weight, physiological effects, and life span. (A)** After post-natal day (PND) 23, the body weight of oligodendrocyte progenitor cell (OPC)-injected twitcher mice (GALC^twi/twi^) and saline-injected twitcher mice (GALC^twi/twi^) decreased compared to the wild type. There was no statistically significant difference in the body weight between OPCs injected twitcher mice (GALC^twi/twi^) and saline injected twitcher mice (*P* >0.05). **(B)** The stride length of the wild type was 6.13 ± 0.15 cm at PND 41. At PND 26, stride length of OPC-injected twitcher mice (GALC^twi/twi^) and saline-injected twitcher mice (GALC^twi/twi^) reached the maximum value, which shortened as the disease progressed. The differences were not statistically significant between the two groups (*P* >0.05). **(C, D)** Twitcher mice (GALC^twi/+^) never twitch at all. OPC-injected twitcher mice (GALC^twi/twi^) and saline-injected twitcher mice (GALC^twi/twi^) had severe twitching at PND 26, and there were no significant differences in the scores of twitching frequency and severity (*P* >0.05). **(E)** A Kaplan-Meier survival analysis using the log-rank test revealed that OPC transplanted mice and control homozygous twitcher mice did not survive beyond 40 days of age. There was no significant difference in life span of the twitcher mice between those that were transplanted with OPCs and saline injection (*P* >0.01). GALC, galactocerebrosidase.

Body weight was an indicator of disease severity. After PND 23, the body weight of twitcher mice (GALC^twi/twi^) with OPC injection or saline decreased compared with wild-type twitcher mice (GALC^twi/+^). The maximum body weights of twitcher mice (GALC^twi/+^) and twitcher mice with OPC injection (GALC^twi/twi^) or saline were 22.03 ± 1.78 g, 6.67 ± 0.06 g, and 6.90 ± 0.10 g, respectively. There was no statistically significant difference in the body weight between twitcher mice (GALC^twi/twi^) with OPC injection and saline injection (*P* >0.05) (Figure 5A).

Gait analysis was performed by measureing the hind stride length. The stride length of twitcher mice (GALC^twi/+^) was 6.13 ± 0.15 cm at PND 41. The maximum stride lengths of twitcher mice (GALC^twi/twi^) with OPC injection or saline were 5.3 ± 0.14 cm and 5.2 ± 0.12 cm at PND 26 (*P* >0.05) (Figure 5B), which shortened as the disease progressed. At PND 38, the stride lengths of twitcher mice (GALC^twi/twi^) with OPC injection or saline were 2.38 ± 0.1 cm and 2.50 ± 0.08 cm (*P* >0.05) (Figure 5B).

The twitching frequency and severity of twitcher mice were observed. Twitcher mice (GALC^twi/+^) never twitch at all. Twitcher mice (GALC^twi/twi^) with OPC injection or saline began severe twitching at PND 26, and there were no significant differences in the scores of twitching frequency and severity (maximum twitching frequency score: 3 ± 0 versus 3 ± 0; maximum twitching severity score: 4 ± 0 versus  ± 0; both *P* >0.05) (Figure 5C and 5D).

To evaluate the effects of OPC transplantation on the survival of twitcher mice, a Kaplan-Meier survival analysis using the log-rank test revealed that transplanted OPCs and control homozygous twitcher mice did not survive beyond 40 days of age. The life span of twitcher mice with OPC injection was 36.25 ± 1.98 days, and the life span in the control group was 36 ± 2.52 days; there was no significant difference in the life span of twitcher mice transplanted with OPCs (*P* >0.01) (Figure 5E). The twitcher mice transplanted with OPCs did not display statistically significant improvements in body weight and behavioral deficits during the post-transplantation survival period compared with control animals.

## Discussion

ESCs can differentiate into various cell types *in vitro*, including neural lineage cells [19-22]. Additionally, because of their self-renewal capacity and pluripotency, ESCs have received increased attention in regenerative medicine and tissue engineering. ESCs may also be a source for cellular replacement for neural degenerative diseases. GLD is caused by mutation(s) in the GALC gene [1-3]. In the absence of GALC activity, psychosine accumulates, and this process appears to account for much of the pathology of GLD, including the loss of oligodendrocytes and diffuse demyelination. Because of the severe deficiency of GALC activity in affected mice, the twitcher mouse is considered to be a valuable model for clinical trials for the treatment of Krabbe disease. Therapeutic approaches, such as bone marrow transplantation, umbilical cord blood transplantation, viral gene transfer of GALC, mesenchymal stem cells, and NSCs [24,25], have been used in the treatment of Krabbe disease but with only partial success. ESC-derived oligodendrocytes may be a source for cell transplantation to treat GLD. In this study, mouse ESC-derived OPCs were transplanted into twitcher mice to assess their therapeutic effects on the disease.

A multi-step protocol was applied for inducing G-Olig2 ESCs into oligodendrocyte [27]. G-Olig2 ESCs is a mouse ESC line with eGFP inserted into the Olig2 gene, a lineage-specific transcription factor for oligodendrocyte differentiation [27,31], which allows for the visualization and separation of oligodendrocytes during the differentiation procedure. With this protocol, oligodendrocyte-specific markers were expressed in G-Olig2 ESC-derived oligodendrocytes at the mRNA level and protein level. A small number of cells expressed astrocyte and neuron markers. Most of the differentiated cells were eGFP-positive. The results indicated that a high percentage of oligodendrocyte-like cells could be derived from G-Olig2 ESCs with this differentiation procedure, as has been reported by other studies [27,31]. With this method, the eGFP-positive oligodendrocytes can be isolated by FACS to remove other cells before cell transplantation.

Psychosine is a toxic metabolite that accumulates in GLD because of the deficiency of GALC activity [32-34]. Normal tissues have very low levels of psychosine. The accumulated psychosine results in subsequent apoptosis of oligodendrocytes and demyelination in human patients and animal models [35,36]. We tested the toxicity of psychosine on differentiated cells and GALC activity before cells were transplanted. The results of toxicity experiments indicated that OPCs were more resistant to psychosine toxicity than terminally differentiated oligodendrocytes. The reason for this resistance is not yet clear. Because the terminally differentiated oligodendrocytes did not survive well, even in low psychosine concentrations, OPCs were chosen as cell transplantation candidates in twitcher mice. Other studies have also found that NSCs are intrinsically resistant to psychosine, but the reason is unknown [37]. GALC activity of OPCs and terminally differentiated oligodendrocytes was approximately 100 times higher compared with TwS1 cells, and there was no difference between OPCs and terminally differentiated cells. GALC activity of these cells was high, and another study reported that GALC activity of bone marrow mesenchymal stem cells and adipose tissue mesenchymal stem cells was only five times higher [12]; neural stem/progenitor cells had 108 nmol/hour per mg protein [38]. Although these cells exhibited an increase in GALC activity, there was no increase in GALC activity in the brains of twitcher mice that received OPC injection. Other studies also found that the GALC activity did not increase after cells were transplanted into twitcher mice brains [12,38]. These cells did not appear to confer any endogenous GALC enzyme to the surrounding cells, as there was no increase in GALC activity in the brains of twitcher mice.

Our experiment demonstrated that the life span and clinical course of twitcher mice injected with OPCs did not improve. The reasons underlying the limited therapeutic efficacy of OPC transplantation are uncertain. Our data indicated that the cells did not survive long; fewer cells were detected after transplantation as time passed. In our *in vitro* experiment, terminally differentiated oligodendrocytes did not survive well even in low-psychosine concentrations. Our data showed that, after transplantation, OPCs differentiated into MBP-expressed cells, which might not withstand the toxicity of psychosine. Some studies have shown that the serum concentration of psychosine increased significantly with the progression of the disease [39]. It was reported that marked accumulation of psychosine was noted in the nervous tissues of the twitcher strain, even on PND 4 (764 ng/100 mg versus 21.6 to 37.2 ng/100 mg wet weight in the nervous tissues of normal mice) [40]. So even very early after birth, the high level of psychosine is already toxic enough to cause cell injury. In twitcher mice, the cell replacement therapy may be improved by more cells and multi-site injection at an early time. However psychosine level significantly increased since PND 4 and the injected cells could not stand. How to increase GALC enzyme level and lower psychosine level is the key point of successful cell replacement therapy.

In our study, twitcher mice did not display significant clinical improvement after cell transplantation. Aside from poor cell survival, another potential explanation was limited migration ability. Our experiment showed that the cells were close to the needle track after transplantation. Even the cells expressed MBP and had remyelin potential, the ability to repair demyelin of the whole CNS was still low because of limited migration ability.

## Conclusion

Our results suggest that, because of poor survival ability and limited migration ability, ESC-derived OPC transplantation is not sufficient to reverse the clinical course in twitcher mice.

## Abbreviations

CNS: central nervous system
DAPI: 6-diamidino-2-phenylindole
DMEM: Dulbecco’s modified Eagle’s medium
EB: embryoid body
eGFP: enhanced green fluorescent protein
ESC: embryonic stem cell
FACS: fluorescence-activated cell sorting
FSG: fish skin gelatin
GALC: galactocerebrosidase
GAPDH: glyceraldehyde-3-phosphate dehydrogenase
GLD: globoid cell leukodystrophy
LIF: leukemia inhibitory factor
MBP: myelin basic protein
NeuN: neuronal nuclear antigen
NPC: neural progenitor cell
NSC: neural stem cell
OEC: olfactory ensheathing cell
OPC: oligodendrocyte progenitor cell
PBS: phosphate-buffered saline
PCR: polymerase chain reaction
PDGF: platelet-derived growth factor
PFA: paraformaldehyde
PND: post-natal day
RT: reverse transcription
RT-PCR: reverse transcription-polymerase chain reaction
SD: standard deviation
